# Interfacial AIE for Orthogonal Integration of Holographic and Fluorescent Dual‐Thermosensitive Images

**DOI:** 10.1002/advs.202105903

**Published:** 2022-02-03

**Authors:** Ye Zhao, Haiyan Peng, Xingping Zhou, Zhong'an Li, Xiaolin Xie

**Affiliations:** ^1^ Key Lab for Material Chemistry of Energy Conversion and Storage Ministry of Education School of Chemistry and Chemical Engineering and National Anti‐Counterfeit Engineering Research Center Huazhong University of Science and Technology (HUST) Wuhan 430074 China; ^2^ Key Lab for Material Chemistry of Energy Conversion and Storage Ministry of Education Hubei Key Laboratory of Material Chemistry and Service Failure School of Chemistry and Chemical Engineering HUST Wuhan 430074 China

**Keywords:** aggregation induced emission, anticounterfeiting, holography, liquid crystals, thiol‐ene click reaction |orthogonality

## Abstract

Orthogonal integration of thermosensitive images is of vital significance for advanced anticounterfeiting, which however remains formidably challenging due to the trade‐off that facile thermoresponse needs easy molecular motion while robust imaging requires molecular restriction. Herein, a viable approach is demonstrated to tackle the challenge by in situ fixing a predesigned aggregation induced emission luminogen (AIEgen) at the polymer/liquid crystal (LC) interface via precisely controlled interfacial engineering, in which the AIEgen is enriched in LC phases during polymerization induced phase separation and subsequently driven to the interface by the interfacial thiol‐ene click reaction. Crosstalk‐free integration of holographic and fluorescent dual‐thermosensitive images with high sensitivity, high contrast ratio, and robust performance is successfully realized in a single unit, attributed to the simultaneously LC‐facilitated AIEgen molecular motion and polymer‐restricted AIEgen diffusion at the interface. The exciting characteristics of these orthogonally integrated dual images will enable them to prevent illegal replication and thus are expected to be promising for high‐security‐level anticounterfeiting applications.

## Graphical abstract

A novel type of advanced materials for security are deliberately designed with the AIEgen chemically fixed at the holographic polymer/liquid crystal interface, based on which the orthogonal integration of high contrast thermosensitive holographic and fluorescent dual images is demonstrated. There dual images show attractive thermoresponsivity and high robustness, promising for high‐security‐level anticounterfeiting applications.

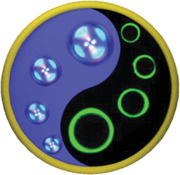

## 1 Introduction

Nowadays, the ever‐increasing forgery has seriously threatened the public security and people's life, forcing us to develop advanced anticounterfeiting materials and technologies.^[^ ^1^ ^]^ Optical security images have an attractive advantage, that is, ease of identification by the public using naked eyes, thereby showing great application prospects. Although a variety of optical images have been produced by holography,^[^ ^2^ ^]^ photonic crystal,^[^ ^3^ ^]^ luminescent materials,^[^ ^1^, ^4^ ^]^ and others,^[^ ^5^ ^]^ simple optical labels have become increasingly inadequate for high‐security‐level anticounterfeiting requirements. Thus, to increase the security, there is an urgent need for the crosstalk‐free integration of different optical images into one element.^[^ ^6^ ^]^

Aggregation induced emission luminogens (AIEgens) recently have drawn particular attention as luminescent anticounterfeiting materials,^[^ ^2^, ^4^, ^7^ ^]^ due to their high emission efficiency in the aggregated state, unlike most traditional luminogens with adverse aggregation‐caused quenching effect. Another attractive feature of AIEgens is that their optical properties can be tuned by external stimuli (e.g., thermal stimulus), thereby gifting the resulting fluorescent label additional security function. More exctingly, Tang et al. very recently demonstrated that the thermosensitivity of AIEgens can be significantly improved by combing with phase change materials (e.g., saturated fatty acids),^[^ ^8^ ^]^ suggesting a useful strategy to reconstruct thermosensitive optical images.

With respect to the phase change materials, liquid crystals (LCs) are particularly important because they can not only exhibit facile phase transition upon heating but also concurrently exhibit fluidity like a liquid and anisotropic properties (e.g., birefringence) like a crystal in aggregation.^[^ ^9^ ^]^ By doping an AIEgen into cholesteric LC microdroplets, Yu et al. have successfully integrated structurally reflective and fluorescent dual images.^[^ ^3a^ ^]^ For the first time, our group have demonstrated that, by doping suitable AIEgens into the polymer/LC composites, the thermal responsiveness can be gifted to the orthogonally integrated holographic and fluorescent dual security images.^[^ ^2a^ ^]^ Nevertheless, to meet advanced security applications, it is highly desirable to explore a comprehensive solution that can simultaneously boost the thermosensitivity, contrast ratio (*CR*) and robustness of thermosensitive images, which indeed remains a significant challenge. Such challenge comes from the following two facts: 1) the aggregation degree of AIEgens is limited in the fluidic LC host, thereby lowering the emission efficiency and reducing the contrast ratio during temperature variation and 2) the physically dispersed AIEgens in the LC host are prone to diffuse randomly, causing poor long‐term stability of the resulting fluorescent images.

**Figure 1:**
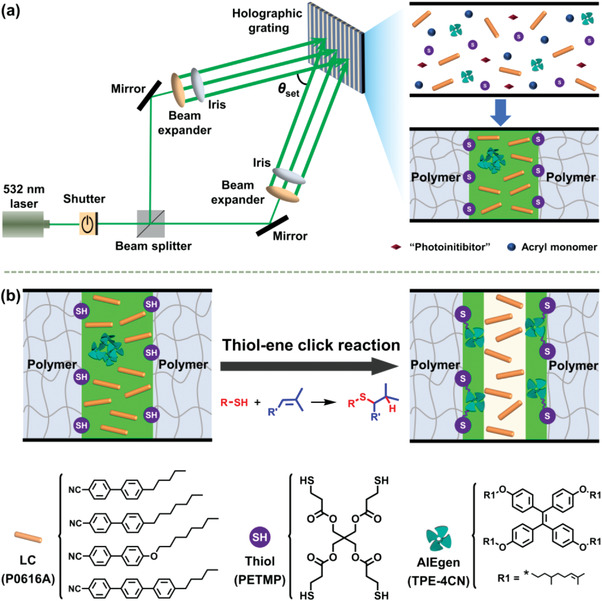
a) Schematic illustration on the construction of thiol‐decorated interfaces by holography and b) the subsequent fixation of the AIEgen at the interface via thiol‐ene click reaction.

Herein, we disclose that chemical fixation of the AIEgen at the polymer/LC interface could not only significantly improve the thermosensitivity and contrast ratio of both fluorescent and holographic images, but also could greatly improve the long‐term stability of these optical security images, which thus provides an efficient solution to the above‐mentioned challenge. As illustrated in **Figure** **1**, the chemical fixation of AIEgen at the polymer/LC interface is implemented in two sequential steps: a) constructing thiol‐decorated polymer/LC interfaces during holographic patterning, wherein the AIEgen can be enriched in LC phases and b) fixing the AIEgen at the interface via the interfacial thiol‐ene click reaction. Therefore, dual‐security images, i.e., holographic and fluorescent images, could be orthogonally integrated, and both of them would show highly sensitive cooperative‐thermoresponse because of the maintained synergy of the AIEgen with the LC,^[^ ^2a^ ^]^ which can provide a new paradigm for designing advanced anticounterfeiting materials.

## 2 Results and Discussion

### 2.1 Chemical Fixation of the AIEgen at the Holographic Polymer/LC Interface

To demonstrate the proof of our concept, thiol‐decorated interfaces were first constructed during holographic patterning, which then underwent thiol‐ene click reaction to fix the AIEgen at the holographic polymer/LC interface. Holographic patterning represents a unique technique to reconstruct the whole information of light waves, which can be implemented via photopolymerization induced phase separation when irradiated by laser interference patterns.^[^ ^2b,c^ ^]^ Generally, alternative bright and dark regions can be generated when two coherent laser beams interfere. When reconstructing holographic polymer/LC composites from the homogeneous monomer/LC mixture, photopolymerization of monomers would occur in the bright regions while the LC is squeezed into the dark regions, thereby producing holographic gratings with alternative polymer and LC phases (Figure 1a). Herein, transmission type and nonslanted holographic gratings (thickness: 10 µm) were fabricated since the bisector of the two coherent laser beams is normal to the sample surface. In addition, the grating pitch was designed to be ≈940 nm by the external angle (33°) and wavelength (532 nm) of these two laser beams according to Bragg's law.^[^ ^2a,b,d,g^ ^]^ Note that a “photoinitibitor” comprising of rose Bengal and *N*‐phenylglycine was used to mediate the holographic patterning based on our previous work,^[^ ^2d,g^ ^]^ which can enable negligible background to the AIEgen's emission after proper photobleaching treatment.^[^ ^2a^ ^]^

Moreover, a multithiol compound was added into the homogeneous acryl monomer/LC mixtures (Entry 6 in Table S1, Supporting Information), which can participate into the photopolymerization during holographic patterning and further produce thiol‐decorated interfaces due to the residual mercaptofunctional groups. Subsequently, the thiol‐ene click reaction was used to fix the AIEgen at the interface considering the high chemical selectivity, high regioselectivity and quantitative kinetics even for low reactivity alkenes (e.g., citronellyl derivatives, Figure S2, Supporting Information).^[^ ^2^, ^10^ ^]^ Herein, the photomediated thiol‐ene click reaction was implemented upon flood green light irradiation (peak wavelength: 512 nm; full‐width at half‐maximum, i.e., FWHM: 33 nm; intensity: 5 mW cm^−2^). During the 48 h of photoreaction, the “photoinitibitor” was also completely photobleached.

It is worth noting that all elements including the LC, thiol compound and AIEgen were well thought out in our design (Figure 1b). P0616A is used as the LC due to its nematic feature and large birefringence (*∆n* = 0.2) at room temperature, as well as suitable phase transition temperature (e.g., *T*_NI_ = 331 K). Pentaerythritol tetra(3‐mercaptopropionate) (PETMP) is selected as the thiol compound given that more mercaptofunctional groups are expected to remain at the interface after holographic photopolymerization. 1,1,2,2‐Tetrakis(4‐((3,7‐dimethyloct‐6‐en‐1‐yl)oxy)phenyl)ethene, namely TPE‐4CN, is prepared as the AIEgen based on three main considerations: 1) It can be enriched in LC phases during holographic patterning due to its high compatibility with the nematic LC but poor miscibility with acryl monomers and polymers,^[^ ^2a^ ^]^ and its diffusion to the interface could be facilitated by the fluidic LC during the subsequent interfacial thiol‐ene click reaction. 2) Citronellyl functional groups have a certain click reaction activity (Figure S4, Supporting Information). 3) Tetraphenylethylene core can undergo photocyclization upon UV irradiation to quench fluorescence (Figure S5, Supporting Information), thereby enabling fluorescent patterning within holographic polymer/LC composites.^[^ ^2a^ ^]^

### 2.2 Facile Thermoresponse Enabled by Interface Engineering

**Figure 2:**
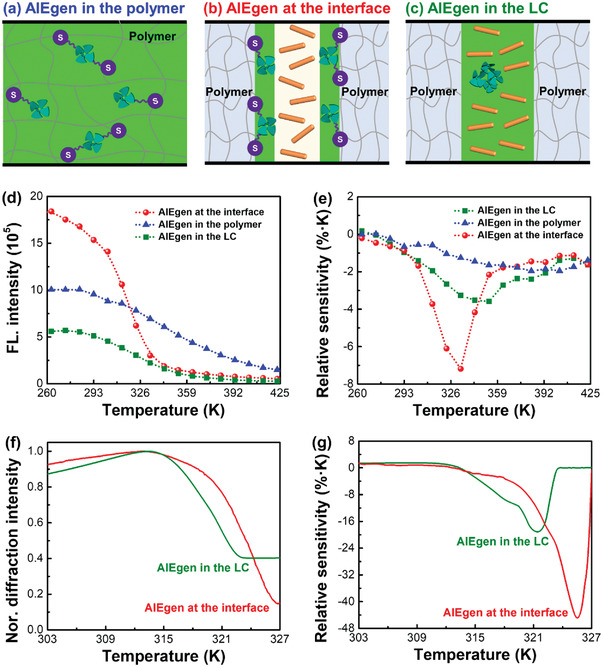
Boosted thermosensitivity and contrast ratio by fixing the AIEgen at the holographic polymer/LC interface. Schematic illustration of the AIEgen when a) crosslinked in the polymer matrix, b) bonded at the polymer/LC interface, and c) enriched in the LC phase. d) Maximum fluorescence (FL.) intensity and e) relative sensitivity against temperature when the AIEgen was crosslinked in the polymer matrix, bonded at the interface and enriched in the LC phase, respectively. Excitation wavelength: 380 nm. f) Normalized (Nor.) diffraction intensity and g) relative sensitivity of the holographic gratings against temperature when the AIEgen was bonded at the polymer/LC interface and enriched in the LC phase, respectively. Sample thickness: 10 µm.

Due to the above careful design, high thermosensitivity and contrast ratio were achieved simultaneously for both fluorescent and holographic functions. As a comparison, we further examined the thermoresponse of these functions with the AIEgen enriched in the LC or crosslinked in the polymer matrix. Schematic illustration of the material design is displayed in **Figure** **2**a–c. Note that the AIEgen content (3 wt%) and sample thickness (10 µm) were controlled at the same level in all systems.

For the fluorescent performance, when the AIEgen was chemically bonded at the polymer/LC interface, a high photoluminescence quantum yield of 35% can be achieved for the holographic polymer/LC composites, and the emission intensity (1.8 × 10^6^) at 263 K is 3.3 and 1.8 times larger than those for the AIEgen enriched in the LC and crosslinked in the polymer matrix, respectively (Figure 2d). The enhanced emission intensity of the AIEgen when fixed at the interface can be simultaneously ascribed to two main reasons: 1) the intramolecular motions of the AIEgen are greatly restricted by the crosslinked polymer at the interface;^[^ ^11^ ^]^ 2) more light energy is gained by the AIEgen due to efficient energy transfer from the LC to the AIEgen^[^ ^2a^ ^]^ and high light scattering of the nematic LC.^[^ ^12^ ^]^ In addition, the *CR* (35) of thermoresponse, defined as the ratio of maximum to the minimum intensity,^[^ ^13^ ^]^ *CR* = *I*_max_/*I*_min_, was increased 1.7 and 5.2 times when fixing AIEgen at the interface, compared with those with the AIEgen enriched in the LC and crosslinked in the polymer matrix, respectively.

More impressively, the maximum relative sensitivity (*S*_r_^max^, −7.2% K^−1^, Figure 2e)^[^ ^8^, ^14^ ^]^ during thermoresponse is significantly boosted by fixing the AIEgen at the interface. According to the definition of relative sensitivity,^[^ ^14^ ^]^ *S*_r_ = (1/*I*)·(∂*I*/∂*T*), *S*_r_ is highly dependent on the temperature and can approach the *S*_r_^max^ at 333 K. This temperature is very close to the nematic‐isotropic phase transition temperature (331 K) of the LC, indicating the facilitation of molecular motions of the AIEgen by the LC. Interestingly, the achieved *S*_r_^max^ is not only 3.8 times higher than that when the AIEgen was crosslinked in the polymer matrix, but also 2.0 times larger than that when the AIEgen was enriched in the LC.

For holographic performance, the thermoresponse is mainly due to the refractive index decrease of the LC during nematic‐isotropic phase transition (Figure 2f).^[^ ^2a^ ^]^ The presence of thiol groups can increase the refractive index of polymer regions,^[^ ^15^ ^]^ which will decrease the refractive index difference between the polymer and LC phases at elevated temperatures and thus enables a higher *CR* of thermoresponse. When fixing the AIEgen at the interface, the *CR* (6.8) is 2.7 times higher than the counterpart when enriching the AIEgen in the LC, and the *S*_r_^max^ (−44.5% K^−1^@326 K, Figure 2g) is also 2.3 times higher. In sharp contrast, the thermoresponse of holographic performance is negligible when the AIEgen is crosslinked in the polymer network due to the absence of LC.^[^ ^16^ ^]^

### 2.3 Long‐Term Stability of Fluorescent Images via Interface Engineering

**Figure 3:**
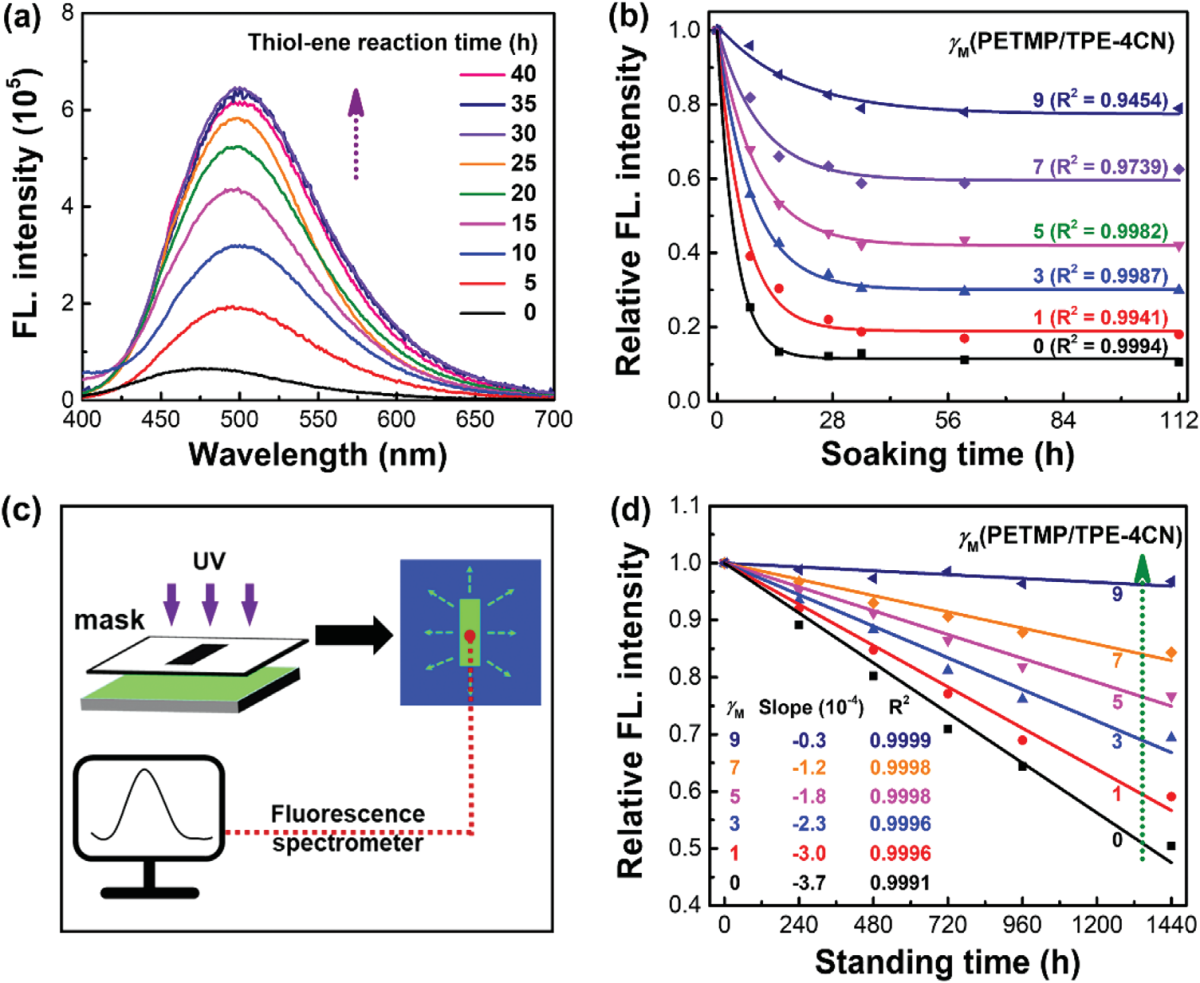
Boosting the fluorescence stability by chemically fixing the AIEgen at the holographic polymer/LC interface. a) Fluorescent spectra (excitation@380 nm) of holographic gratings after interfacial thiol‐ene click reaction with different time and being soaked in *n*‐hexane for 48 h. *γ*_M_(PETMP/TPE‐4CN) was 9. b) Relative fluorescence (FL.) intensity (excitation@380 nm) of holographic gratings against soaking time with different *γ*_M_(PETMP/TPE‐4CN). c) Scheme of monitoring the AIEgen diffusion by fluorescent spectroscopy. d) Relative fluorescence (FL.) intensity of the AIEgen in holographic gratings against the standing time under ambient condition, in which no solvent was present. *γ*_M_(PETMP/TPE‐4CN) was varied from 0 to 9. Testing level for statistical analysis: *p* < 0.05.

In addition to the boosted *CR* and *S*_r_^max^, the long‐term stability of fluorescent images is also greatly improved, attributed to the effectively constrained AIEgen diffusion when fixed at the holographic polymer/LC interface. The chemical fixation of the AIEgen was examined by soaking the holographic gratings in *n*‐hexane for 48 h since the LC and unfixed AIEgen will be removed by *n*‐hexane (Figure S7, Supporting Information).^[^ ^2a^ ^]^ **Figure** **3**a displays the fluorescent spectra of the holographic gratings after soaking in *n*‐hexane for 48 h. Clearly, the fluorescent emission gradually increases by up to about tenfold with an augmentation of the thiol‐ene reaction time, and then levels off when the light irradiation time is more than 30 h. Since the emission intensity is linearly proportional to the AIEgen concentration after *n*‐hexane treatment (Figure S8, Supporting Information), the clearly increased emission intensity confirms that ≈10% of the AIEgen was fixed in the polymer‐rich phase during holographic patterning, and ≈90% of the AIEgen was fixed at the holographic polymer/LC interface through the subsequent interfacial thiol‐ene click reaction.

We also found that the chemical fixation of AIEgen at the interface is highly dependent on the functional group ratio of thiol to AIEgen, namely *γ*_M_(PETMP/TPE‐4CN). As illustrated in Figure 3b, the fluorescent emission is gradually decreased and finally levels off with an increase of the soaking time due to the removal of the LC and unbonded AIEgen by *n*‐hexane. However, the emission intensity is significantly increased with an increase of *γ*_M_(PETMP/TPE‐4CN) from 0 to 9, suggesting the increased amount of AIEgen molecules bonded at the interface. Clearly, chemical bonding at the interface can greatly improve the solvent resistance of the AIEgen.

We then investigated the chemical fixation of the AIEgen by designing a rectangular fluorescent pattern and monitoring the emission intensity with standing time under ambient condition (Figure 3c). The fluorescent pattern was fabricated by the photocyclization of AIEgen core.^[^ ^2a^ ^]^ The AIEgen diffusion from unexposed to exposed regions would be possible due to the concentration gradient. As clearly displayed in Figure 3d, the emission intensity linearly decays with the standing time, further confirming the above conclusion. Excitingly, the emission decay can be effectively constrained when increasing *γ*_M_(PETMP/TPE‐4CN), and it is negligible at the *γ*_M_(PETMP/TPE‐4CN) of 9.

### 2.4 Direct Imaging of the AIEgen Decorated Polymer/LC Interface

The chemical fixation of AIEgen at the polymer/LC interface was further confirmed by polarized optical microscopy and confocal microscopy. Given that the LC domains within the holographic gratings were too small (277 ± 69 nm) to identify by these two optical microscopy methods, we fabricated polymer/LC composites with larger LC droplets via anionic reaction induced phase separation. Anionic reactions between thiol and acryl monomers were confirmed by high‐resolution mass spectroscopy (HRMS, Figures S11–S14, Supporting Information).

**Figure 4:**
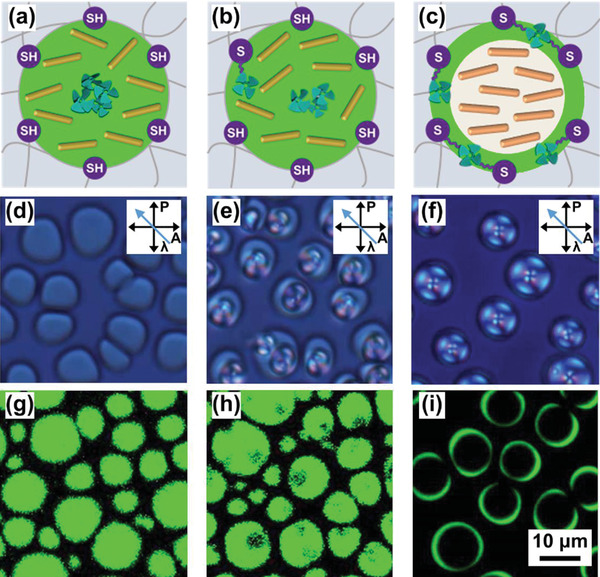
Tuning the spatial location of the AIEgen from the LC phase to the polymer/LC interface via interfacial thiol‐ene click reaction. a–c) Schematic illustration of the spatial location of the AIEgen when increasing the time of thiol‐ene click reaction. d–f) Polarized optical microscopy and g–i) confocal microscopy images of polymer/LC composites. The thiol‐ene reaction was exerted under green light (peak wavelength: 512 nm, FWHM: 33 nm, intensity: 5 mW cm^−2^) and the reaction time was d,g) 0 min, e,h) 10 min, and f,i) 48 h, respectively. The LC droplet size was 8.7 ± 2.0 µm (sample size: n = 50). The polymer/LC composites were formed via anionic reaction induced phase separation for 24 h in dark prior to thiol‐ene click reaction. The fluorescent emission was excited by 405 nm light to prevent the LC excitation. The imaging parameters were adjusted under optimized conditions for each sample. *γ*_M_(PETMP/TPE‐4CN) was 9.

Since the thiol‐based anionic reaction is orthogonal to the radical‐mediated thiol‐ene click reaction,^[^ ^2^, ^10^ ^]^ the AIEgen with citronellyl functional groups could not participate into the anionic reaction and then would be enriched in the LC phases during phase separation, enabling us to directly monitor the AIEgen diffusion from the LC phases to the polymer/LC interface during the interfacial thiol‐ene click reaction (**Figure** **4**a–c). As illustrated in Figure 4d,g, the LC droplets in a size of 8.7 ± 2.0 µm, which were formed at the anionic reaction time (ART) of 24 h, displayed bright lime‐green emission when excited by 405 nm light, while the LC cannot be excited at this wavelength.^[^ ^2a^ ^]^ Negligible emission of the polymer regions indicates that the AIEgen is primarily enriched in the LC phases during phase separation, although the nematic texture is unclear. In contrast, nematic textures of the LC droplets become clearer upon flood green light irradiation (Figure 4e,f), suggesting the separation of the AIEgen with the LC. Confocal images clearly show that the AIEgen diffuses from the LC phases to the polymer/LC interface due to the interfacial thiol‐ene click reaction (Figure 4h,i). Note that a multithiol compound is required and the ART should be less than 48 h toward this target (Figures S15–S17, Supporting Information).

### 2.5 Interfacial Engineering via the Synergy of Thiol‐Based Orthogonal Reactions

**Figure 5:**
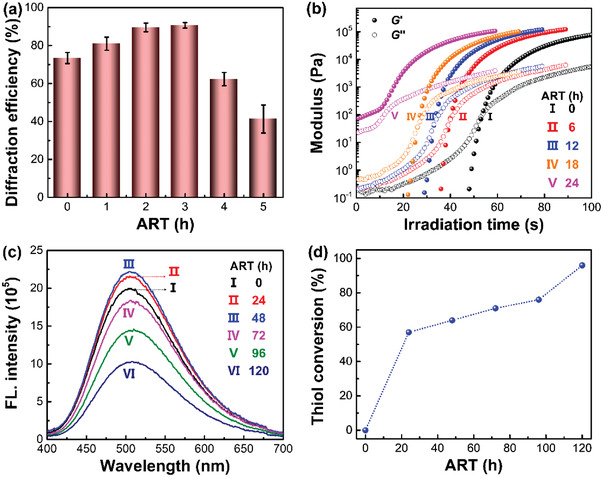
Effects of anionic reaction on the holographic and fluorescent properties. a) Diffraction efficiency of holographic gratings against ART (sample size: *n* = 25). b) Photorheological plots upon exposure to 38 mW cm^−2^ of 420–500 nm light after different time of anionic reaction, where the crossover of storage (*G*') and loss moduli (*G*") was considered as the gel point. c) Fluorescent emission of holographic gratings as a function of ART. d) Thiol conversion against ART. *γ*_M_(PETMP/TPE‐4CN) was 9 and the interfacial thiol‐ene click reaction time was 48 h.

The synergy of thiol‐based orthogonal reactions, that is, anionic reaction and thiol‐ene click reaction, was found to be critical for the precise interfacial engineering. For instance, the holographic performance was found to be highly dependent on the ART. Diffraction efficiency is the primary parameter to evaluate the holographic performance, defined as the intensity ratio of diffraction to the sum of both diffraction and transmission at the Bragg angle (Figure S18, Supporting Information). A 5 mW of 633 nm laser was employed to measure the diffraction efficiency in a nondestructive manner. Results show that the diffraction efficiency is increased 1.2 times (i.e., from 74% ± 3% to 91% ± 1%) when increasing the ART from 0 to 3 h, due to the enhanced phase separation (Figure S19, Supporting Information). Nevertheless, because of the uncontrolled phase separation, the diffraction efficiency is dramatically decreased to 41% ± 7% when further increasing the ART to 5 h (**Figure** **5**a).

Constrained diffusion of thiyl radicals from the bright to dark regions should be the primary reason for the improved holographic performance when increasing the ART from 0 to 3 h. As widely accepted, well‐defined phase separation is highly desired for high performance holographic images.^[^ ^2c,g^ ^]^ However, chain transfer reactions are generally dominant in the thiol‐contained systems,^[^ ^10a,b^ ^]^ and the newly generated thiyl radical at this stage will trigger side polymerizations in the dark region during holographic patterning, thereby causing uncontrolled phase separation. To depress side polymerizations by chain transfer, it is reliable to decrease the diffusion capability of the thiol via anionic reactions. This conclusion is supported by the dramatically improved phase separation and diffraction efficiency when increasing the thiol functionality or decreasing the thiol content (Figures S20–S23, Supporting Information).

The shortened gelation of photopolymerization is responsible for the depressed phase separation when extending ART more than 3 h.^[^ ^2c^ ^]^ As illustrated in Figure 5b, the crossover of storage and loss moduli during the photorheological measurements can be considered as the gel point since the storage modulus is generally lower than the loss modulus before gelation and becomes higher after gelation.^[^ ^2^, ^17^ ^]^ The gelation time of photopolymerization decreased from 54 to 26 s when increasing the ART from 0 to 18 h, and the gelation even occurred before photopolymerization at the ART of 24 h. This is reasonable due to the consumption of both acryl and thiol monomers by anionic reactions. Clearly, an appropriate ART is crucial for boosting the phase separation and holographic performance in the thiol‐contained systems.

Moreover, the fluorescent emission was also found to be highly dependent on the ART. As illustrated in Figure 5c, the emission intensity is increased by 12% when increasing the ART from 0 to 48 h, but significantly decreased if further prolonging the ART. The weakened fluorescent emission could be attributed to the decreased thiol content for fixing the AIEgen at the polymer/LC interface after longer time of anionic reaction, which can be rationalized by the significantly enhanced emission when increasing the thiol content during thiol‐ene click reaction (Figure S24, Supporting Information). This interpretation was further supported by the increased consumption of the thiol functional group from 0% to 94% when increasing the ART from 0 to 120 h (Figure 5d), according to real‐time Fourier transform infrared spectroscopy. Note that the relatively weak emission when the ART was less than 48 h is due to the reaction competition between the thiol‐acryl photopolymerization and thiol‐ene click reaction upon light irradiation. For instance, the conversion of thiol and acryl monomers can approach 99% upon light irradiation at the ART of 0 h, while the conversion of the citronellyl functional group was only 11% due to the relatively low reactivity (Figure S28, Supporting Information). By extending the ART to 48 h, the conversion of citronellyl functional group can be increased significantly upon light irradiation so that more TPE‐4CN molecules can be fixed at the interface.

Clearly, the thiol‐based anionic reaction prior to the interfacial thiol‐ene click reaction plays a key role for simultaneously boosting the holographic performance and fluorescent emission. Both diffraction efficiency and fluorescent emission have been improved by controlling the ART at 3 h when *γ*_M_(PETMP/TPE‐4CN) was 9, and as high as 91% ± 1% of diffraction efficiency was achieved.

### 2.6 Robust Thermoresponse of Dual‐Security Images

**Figure 6:**
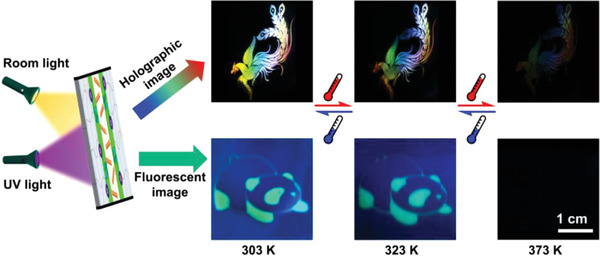
Cooperative‐thermoresponse of holographic and fluorescent dual images when fixing the AIEgen at the polymer/LC interface. The holographic image was viewed under room light while the fluorescent image was recognized under UV light.

Finally, fixing the AIEgen at the holographic polymer/LC interface makes us successfully realize the orthogonal reconstruction of high performance holographic and fluorescent dual security images in a single unit. Holographic images were fabricated by projecting the object information into the laser interference field during holographic patterning.^[^ ^2b^ ^]^ Differently, fluorescent images were patterned under 365 nm UV light via photocyclization of the AIEgen core that changes the emission color from lime‐green to blue.^[^ ^2a^ ^]^ As shown in **Figure** **6**, the holographic image of “phoenix” and fluorescent image of “panda” can be independently identified by the naked eyes under room light and UV light, respectively. These dual images are crosstalk‐free, as the fluorescent image is viewed when the fluorophores (e.g., LC and AIEgen) are only excited by the UV light while the holographic image is only displayed by diffracting the visible light. More importantly, these dual images show reversible cooperative‐thermoresponse. The brightness of both images is significantly weakened when increasing the temperature from 303 to 373 K, which can fully back when the temperature decreases to 303 K. Compared with the common security methods by separating the holographic and fluorescent images, the noninterference integration of dual images in a single element can greatly improve the thermosensitivity of both images ascribed to the synergy of the AIEgen with the LC,^[^ ^2a^ ^]^ and thus presents exclusive advantages to significantly improve the security‐level of anticounterfeiting. When it comes to authentication, one can recognize the publicly visible holographic image under visible light, and then identify the covered fluorescent image under UV light, finally identify the cooperative‐thermoresponse of holographic and fluorescent images.

**Figure 7:**
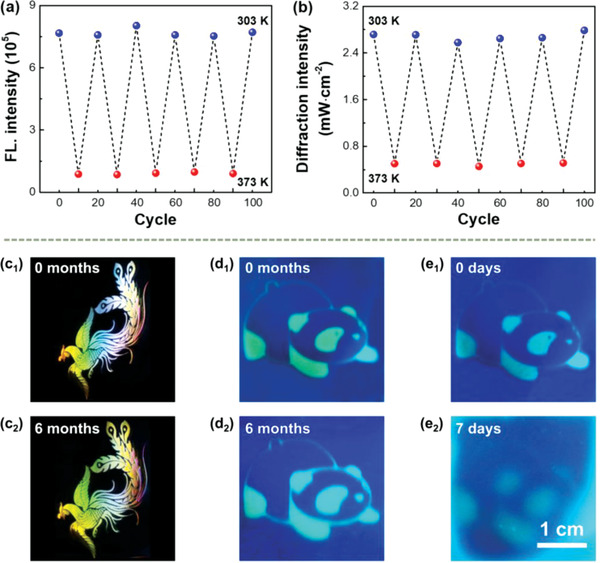
Robust cooperative‐thermoresponse and long‐term stability of holographic and fluorescent dual‐security images. a) Fluorescence (FL) intensity and b) diffraction intensity when varying the temperature between 303 and 373 K. c_1_,c_2_) Holographic images after 0 and 6 months, respectively. d_1_,d_2_) Fluorescent images after 0 and 6 months when fixing the AIEgen at the polymer/LC interface. e_1_,e_2_) Fluorescent images after 0 and 7 d when enriching the AIEgen in the LC phases.

Excitingly, not only facile thermoresponse over 100 cycles of both holographic and fluorescent images was achieved (**Figure** **7**a,b), but also the long‐term stability of fluorescent images was greatly improved. As shown in Figure 7c_1_,c_2_, almost no change of holographic images is noted after 6 months. In addition, there is negligible change observed for the fluorescent image due to the bonding of AIEgen at the holographic polymer/LC interface (Figure 7d_1_,d_2_). In sharp contrast, the fluorescent image significantly blurred after 7 d if enriching the AIEgen in the LC phases (Figure 7e_1_,e_2_), attributed to the random molecular diffusion. The high performance holographic and fluorescent dual images achieved in this work we believe are difficult to replicate using other methods, which thus have a great potential for high‐security‐level anticounterfeiting applications.

## 3 Conclusion

In summary, we demonstrated the first example of high contrast thermosensitive and robust dual‐security images in a single material, i.e., holographic and fluorescent images. Such achievement was enabled by chemically fixing the AIEgen at the polymer/LC interface via interfacial thiol‐ene click reaction, which can be confirmed by polarized optical microscopy and confocal microscopy. Compared with the cases of the AIEgen in the LC phase or polymer phase, the fixation of AIEgen at the polymer/LC interface not only helped to boosting the thermosensitivity and contrast ratio of both holographic and fluorescent images, but also greatly prolonged the image preservation time due to the restricted AIEgen diffusion. Furthermore, the thiol‐based anionic reaction, which is orthogonal to the radical mediated thiol‐ene click reaction, was found to play a key role in simultaneously boosting fluorescent emission and holographic performance, and the reaction time needed to be controlled at 3 h. Furthermore, these crosstalk‐free dual‐security images can encouragingly show highly sensitive and robust cooperative‐thermoresponse, paving a new way toward the design of advanced materials for high‐security‐level anticounterfeiting.

## 4 Experimental Section

### Preparation of Monomer/LC Mixtures

To prepare homogeneous mixtures for holographic patterning, the AIEgen (e.g., TPE‐4CN), LC (e.g., P0616A), “photoinitibitor” composed of rose Bengal (RB), and *N*‐phenylglycine (NPG) were added into the monomer mixture comprising *N,N*‐dimethylacrylamide (DMAA) and 6361–100 (weight ratio: 2/1). Bulk‐ultrasonication was exerted at 313 K to facilitate the formation of homogeneous mixtures. Then, the thiol (e.g., butyl 3‐mercaptopropionate (BMP), ethylene glycol bis(3‐mercaptopropionate) (EGBMP), trimethylolpropane tris(3‐mercaptopropionate) (TMPTMP), and PETMP, respectively) was added into the mixture in dark, followed by magnetic stirring for 5 min in an ice‐water bath. Detailed formulations were listed in Table S1 in the Supporting Information.

### Reconstruction of Holographic Gratings

For holographic patterning, the prepared homogeneous mixtures were added into LC cells (gap: 10 µm) via capillary action. Holographic gratings were fabricated under a 532 nm (*λ*_writing_) coherent laser. In detail, two beams with equal‐intensity were generated by splitting the coherent laser, which were then expanded and well‐collimated, finally irradiated the LC cell with an external angle of ≈33° (*θ*_set_, Figure 1a). The intensity for each beam was 3 mW cm^−2^, and the irradiation time was 3 min. The bisector of these two beams was perpendicular to the cell surface so that unslanted gratings could be achieved. The grating pitch (*Λ*) was calculated to be ≈940 nm according to the Bragg's law,^[^ ^2g^ ^]^ *Λ *= 0.5*λ*_writing_/sin(*θ*_set_/2).

After holographic patterning, interfacial thiol‐ene click reaction was conducted under green light (peak wavelength: 512 nm, FWHM: 33 nm, intensity: 5 mW cm^−2^). All fabricated samples were stored in dark.^[^ ^2a^ ^]^

### Reconstruction of Holographic Images

Holographic images were reconstructed via laser interference, in which the computer‐generated image was projected into the interference field by a spatial light modulator.^[^ ^18^ ^]^ The intensity of each beam was 3 mW cm^−2^ and the irradiation time was 3 min. Finally, interfacial thiol‐ene click reaction was conducted under green light (peak wavelength: 512 nm, FWHM: 33 nm, intensity: 5 mW cm^−2^), during which the residual “photoinitibitor” was photobleached.

### Fluorescent Patterning

Fluorescent images were patterned by UV irradiation (wavelength: 365 nm, intensity: 90 mW cm^−2^) through a photomask. The irradiation time was 5 min. Samples were stored in dark after fluorescent patterning.

### Anionic Reactions

As illustrated in Figure S11 in the Supporting Information, when mixing a thiol compound with an acryl monomer, the thiol is supposed to spontaneously add to the electron‐deficient C═C double bond of the acryl monomer using its lone pair of electrons, producing a zwitterionic intermediate comprising an anionic carbon center. Subsequently, the anionic carbon center would add to a second electron‐deficient C═C double bond, yielding a new anionic carbon center. Repeated addition of acryl monomers in the chain‐growth mechanism is possible until termination occurs via proton transfer, depending on the monomer reactivity. The ART was defined as the period prior to photoreaction when mixing the thiol compound with acryl monomers.

### Statistical Analysis

All plotting and analyses were conducted with the Origin 9.0 software. Experimental results were presented as mean ± SD (standard deviation). Diffraction efficiencies were presented by measuring five independent devices and each device was measured for five times at different sites. Grating depth was shown by measuring ten separate pitches for each sample. LC droplet sizes were displayed by measuring 50 separate droplets. The fluorescence intensity shown in Figure 3b,d, and the diffraction intensity displayed in Figure 2f were normalized to the maximum. Linear regression was conducted in Figure 3d and Figure S8b in the Supporting Information. With respect to the obtained slopes in Figure 3d, *p*‐values were 3.48 × 10^−5^, 3.10 × 10^−5^, 1.25 × 10^−4^, 7.10 × 10^−5^, 1.57 × 10^−4^, and 4.61 × 10^−2^ when *γ*_M_(PETMP/TPE‐4CN) was 0, 1, 3, 5, 7, and 9, respectively. With respect to the obtained slope in Figure S8b in the Supporting Information, the *p*‐value was 7.79 × 10^−8^. According to these *p*‐values, all data were of high reliability.^[^ ^19^ ^]^

## Conflict of Interest

The authors declare no conflict of interest.

## Supporting information

Supporting Information

## Data Availability

The data that support the findings of this study are available from the corresponding author upon reasonable request.
