# Supplementary material for: Interfacial AIE for Orthogonal Integration of Holographic and Fluorescent Dual‐Thermosensitive Images
Source: Adv Sci (Weinh). 2022 Feb 3;9(10):2105903. doi: 10.1002/advs.202105903 (PMC8981879; doi:10.1002/advs.202105903)
Supplement: Supplementary file 1 — Supporting Information [file ADVS-9-2105903-s001.pdf]

ADVANCED SCIENCE
Open Access

Supporting Information

for *Adv. Sci.*, DOI 10.1002/advs.202105903

Interfacial AIE for Orthogonal Integration of Holographic and Fluorescent Dual-Thermosensitive Images

Ye Zhao, Haiyan Peng, Xingping Zhou, Zhong'an Li and Xiaolin Xie**

ADVANCED SCIENCE
Open Access

Supporting Information

for *Adv. Sci.*, DOI: 10.1002/advs.202105903

Interfacial AIE for Orthogonal Integration of Holographic and Fluorescent Dual-Thermosensitive Images

Ye Zhao, Haiyan Peng, Xingping Zhou, Zhong'an Li, Xiaolin Xie**

Supporting Information

Interfacial AIE for Orthogonal Integration of Holographic and Fluorescent Dual-Thermosensitive Images

Ye Zhao, Haiyan Peng, Xingping Zhou, Zhong'an Li, Xiaolin Xie**

Y. Zhao, Prof. H. Y. Peng, Prof. X. P. Zhou, Prof. X. L. Xie

Key Lab for Material Chemistry of Energy Conversion and Storage, Ministry of Education, School of Chemistry and Chemical Engineering, and National Anti-Counterfeit Engineering Research Center, Huazhong University of Science and Technology (HUST), Wuhan 430074, China

E-mail: hypeng@hust.edu.cn (H. Y. Peng), xlxie@hust.edu.cn (X. L. Xie)

Prof. Z. A. Li

Key Lab for Material Chemistry of Energy Conversion and Storage, Ministry of Education, Hubei Key Laboratory of Material Chemistry and Service Failure, School of Chemistry and Chemical Engineering, HUST, Wuhan 430074, China

Contents

1. Experimental Section S3

1.1 Materials S3

1.2 Characterization Methods S4

2. Supplementary Figures S8

2.1 Chemicals S8

2.2 Proposed Mechanism for the Photoreaction of Thiol with Citronellyl S8

2.3 Photoreaction Kinetics of the Thiol/Acryl and Thiol/Citronellyl Systems S9

2.4 Photocyclization of TPE-4CN (the AIEgen) within Holographic Gratings S10

2.5 Comparison of Thermosensitivity S11

2.6 Fluorescent Emission when Fixing the AIEgen at the Interface S11

2.7 Characterization of Anionic Reaction Products S13

2.8 Polarized Optical Microscopy and Confocal Images S15

2.9 Effect of Anionic Reaction Time (ART) on the Holographic Performance S17

2.10 Effect of Thiol Functionality on the Holographic Performance S18

2.11 Effect of Thiol Content on the Holographic Performance S19

2.12 Effect of Thiol Content on the Fluorescent Emission S20

2.13 Effect of ART on the Photoreaction Kinetics S20

3. References S23

1. Experimental Section

1.1 Materials

Rose Bengal (RB, purity: 85%) was purchased from Acros Organics. Ethylene glycol bis(3-mercaptopropionate) (EGBMP, purity: 97%), pentaerythritol tetra(3-mercaptopropionate) (PETMP, purity: 90%) and *N*,*N*-dimethylacrylamide (DMAA, purity: 98%) were received from TCI Chemicals. Butyl 3-mercaptopropionate (BMP, purity: 99%) was obtained from Weng Jiang reagent Co., Ltd., China. Trimethylolpropane tris(3-mercaptopropionate) (TMPTMP, purity: 85%), methyl acrylate (MA, purity: 97%), butyl methacrylate (BMA, purity: 97%) and citronellol (purity: 97%) were acquired from Energy Chemical Co., Ltd., China. Nematic liquid crystal (LC) in the brand name of P0616A ($n_{o\ (589\ nm,\ 293\ K)}$ = 1.52, $n_{e\ (589\ nm,\ 293\ K)}$ = 1.72) was obtained from Shijiazhuang Chengzhi Yonghua Display Material Co., Ltd., China. *N*-Phenylglycine (NPG, purity: 97%) and trimethylolpropane ethoxylate triacrylate (TMPEOTA, average molecular weight: ~692 $g \cdot mol^{-1}$) were purchased from Aladdin. The hyperbranched acrylate monomer with 8 C=C double bonds, namely 6361-100, was donated as a gift by Eternal Chemical Co., Ltd., China. 1,1,2,2-Tetrakis(4-((3,7-dimethyloct-6-en-1-yl)oxy)phenyl)ethene (shorted as TPE-4CN) with 4 citronellyl functional groups was synthesized according to our previous work.[1] Dimethyl sulfoxide (DMSO, AR), *N*,*N*-dimethylformamide (DMF, AR) and *n*-hexane (AR) were acquired from Sinopharm Chemical Reagent Co., Ltd., China. All materials were used directly without further purification.

Table S1. Formulations for holographic patterning[a)]

Entry	Acryl monomers [wt%][b)]	Thiol monomer [wt%]	P0616A [wt%]	TPE-4CN [wt%]	Thiol monomer used	Thiol functionality	Functional group ratio of thiol monomer to TPE-4CN[c)]
1	67.0	0.0	30.0	3.0	/	/	0
2	65.5	1.5			PETMP	4	1
3	62.4	4.6			PETMP	4	3
4	59.3	7.7			PETMP	4	5
5	56.2	10.8			PETMP	4	7
6	53.3	13.7			PETMP	4	9
7	48.4	18.6			BMP	1	9
8	53.5	13.5			EGBMP	2	9
9	52.0	15.0			TMPTMP	3	9

a): The contents of RB and NPG were 1.0 and 1.3 wt%, respectively, relative to the total mass of acryl monomers, thiol monomer, P0616A and TPE-4CN.

b): Acryl monomers were composed of DMAA and 6361-100 (weight ratio: 2/1).

c): Functional group ratio of PETMP to TPE-4CN was denoted as γ_M(PETMP/TPE-4CN) in the text.

1.2 Characterization Methods

(1) High Resolution Mass Spectrometry (HRMS)

To identify the products of thiol-based anionic reaction, HRMS was performed on a mass spectrometer (Compact TOF, Bruker, Germany) that was equipped with an electrospray ionization source. DMAA, MA and BMA were used as mono-acryl monomers, which were homogeneously mixed with a monothiol compound (e.g., BMP), respectively. Then the reaction was maintained at 333 K for 4 days, in which the functional group ratio of acryl to thiol was 4. To be noted, a polar solvent (e.g., DMSO) was needed during the anionic reaction of MA (or BMA) with BMP.

(2) Real-Time Fourier Transform Infrared (RT-FTIR) Spectroscopy

Photoreaction kinetics was characterized on a real-time Fourier transform infrared spectrometer (Vertex 80, Bruker, Germany) with a KBr beam splitter and a mercury cadmium telluride (MCT) detector. Kinetic data were collected every 0.24 s. A light source (S2000, Omnicure, Canada) was employed for triggering the photoreaction and the light wavelength was confined to be 420~500 nm by a filter. Additionally, the light intensity was set as 38 $mW{\cdot}cm^{-2}$. Homogeneous mixtures for photoreaction were sandwiched between two NaCl plates that were then placed onto a horizontal transmission apparatus. The mixture thickness was controlled to be 25 μm by plastic spacers. The conversion of thiol, acryl and citronellyl functional groups was calculated by monitoring the change of absorption peak in the range of 2450~2600, 915~1015 and 820~840 cm^{-1}, respectively,[2]

$$\alpha = 1 - A_t / A_0 \tag{1}$$

where, α was the functional group conversion, A_t and A_0 represented absorption areas at the irradiation time t and before light irradiation, respectively.

To understand the effect of anionic reaction time (ART) on the photoreaction kinetics, 70 wt% of monomers and 30 wt% of LC (e.g., P0616A) were homogeneously mixed, into which the “photoinitibitor” composed of RB and NPG (1.0 and 1.3 wt% relative to the total mass of monomers and LC, respectively) was added to mediate the photoreaction subsequent to anionic reaction.

(3) Rheology

Photorheological behaviors were analyzed using a rotational rheometer (MCR 302, Anto-Parr, Austria). Two parallel plates in a diameter of 25 mm were used to sandwich the sample, in which the above one was metallic while the bottom one was transparent polyester to allow for light passing through. A light source (S2000, Omnicure, Canada) was employed for triggering the photoreaction and the wavelength was confined to be 420~500 nm by a filter. Experiments were isothermally implemented in the strain-sweep mode, in which the normal force, frequency, strain amplitude and chamber temperature were set as 0 N, 1 Hz, 1% and 298 K, respectively. After being sheared for 60 s, the sample in a thickness of 0.1 mm was irradiated by 38 $mW\cdot cm^{-2}$ of 420~500 nm light. The chamber was continuously purged with gaseous nitrogen at a flow rate of 50 $mL\cdot min^{-1}$ to prevent oxygen inhibition. Both storage and loss moduli were simultaneously recorded and their crossover was considered as the gel point.[3]

(4) Characterization of Diffraction Properties

Diffraction efficiencies and temperature-dependent diffraction intensity were characterized using an LC display parameter tester (LCT-5016C, North LC Engineering Research and Development Center, China). A 633 nm laser (5 mW, HNL 050L, Thorlabs, USA) was used to probe the gratings at the Bragg angle, and the diffraction efficiency was defined to be the ratio of diffraction intensity to the sum of diffraction and transmission

intensities at the Bragg angle.[4]

(5) Micromorphology

Micromorphology of holographic gratings was characterized by atomic force microscopy (AFM, SPM-9700, Shimadzu, Japan). Prior to characterizations, the LC in the gratings was removed by immersing samples in *n*-hexane for 48 h.[5] During AFM measurement, the tapping mode was employed with a resonance frequency of 300 kHz.

Morphologies of LC droplets in the polymer/LC composites were characterized by polarized optical microscopy (POM, Axio Scope. A1, Carl Zeiss, Germany), taking advantage of the LC birefringence. Spatial distribution of the AIEgen in the polymer/LC composites was investigated by laser confocal microscopy (FV1000, Olympus, Japan) due to the distinct fluorescent emission. The excitation wavelength was set to be 405 nm at which the LC would not be excited. The sample thickness was controlled to be 3 μm during POM and confocal characterizations.

(6) Fluorescence Spectra

Fluorescence spectra at room temperature were acquired on a fluorescence spectrometer (RF-5301PC, Shimadzu, Japan). Temperature-dependent fluorescence spectra were obtained using another fluorescence spectrometer (FLS1000, Edinburgh Instruments, United Kingdom) that was equipped with a temperature-control apparatus (Optistat DN, Oxford, United

Kingdom). The temperature range and heating rate were set as 263~423 K and 2 K·min^{-1}, respectively. Data were captured every 10 K, and the system was isothermally maintained for 5 min prior to capturing the data. The sample thickness was set as 10.0 ± 0.5 μm.

2. Supplementary Figures

2.1 Chemicals

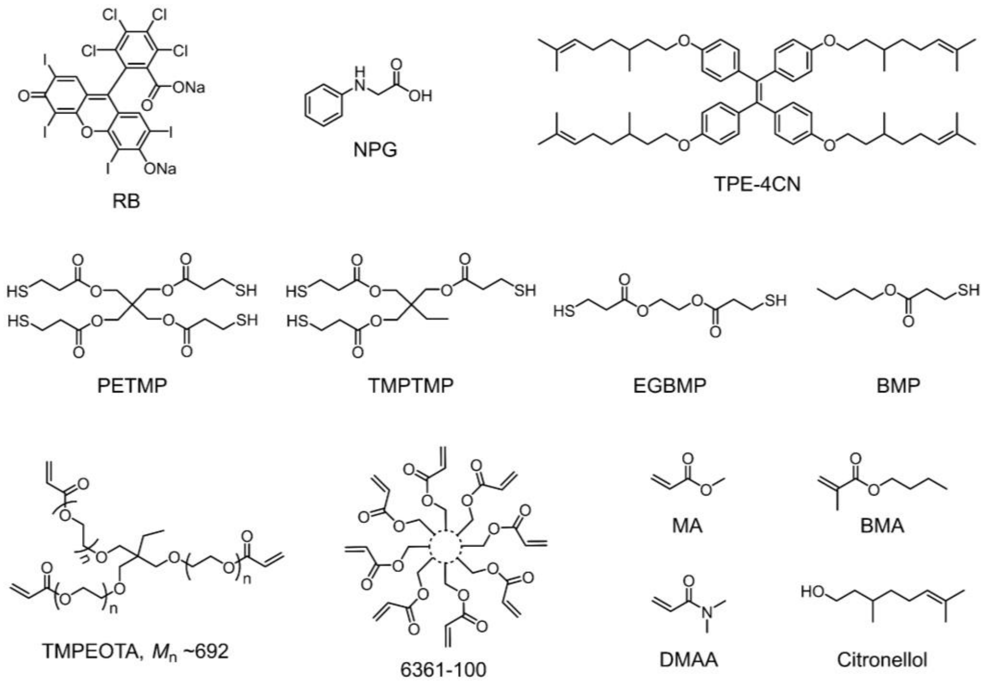

Figure S1. Chemical structures of RB, NPG, TPE-4CN, PETMP, TMPTMP, EGBMP, BMP, TMPEOTA, 6361-100, MA, BMA, DMAA and citronellol.

2.2 Proposed Mechanism for the Photoreaction of Thiol with Citronellyl

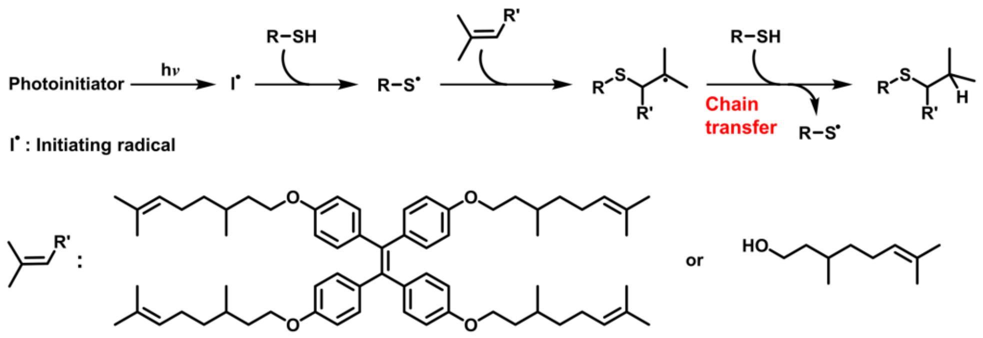

Figure S2. Proposed mechanism for the photoreaction of thiol with citronellyl. The

photoreaction is thiol-ene click reaction.[2b,6] A thiyl radical can be produced when the initiating radical abstracts a hydrogen atom from the thiol, which then would add to the C=C double bond of the alkene that is substituted with two methyl groups. Subsequently, the generated carbon-centered radical reacts with another thiol via chain transfer reaction, yielding the thioether product and a new thiyl radical.

2.3 Photoreaction Kinetics of the Thiol/Acryl and Thiol/Citronellyl Systems

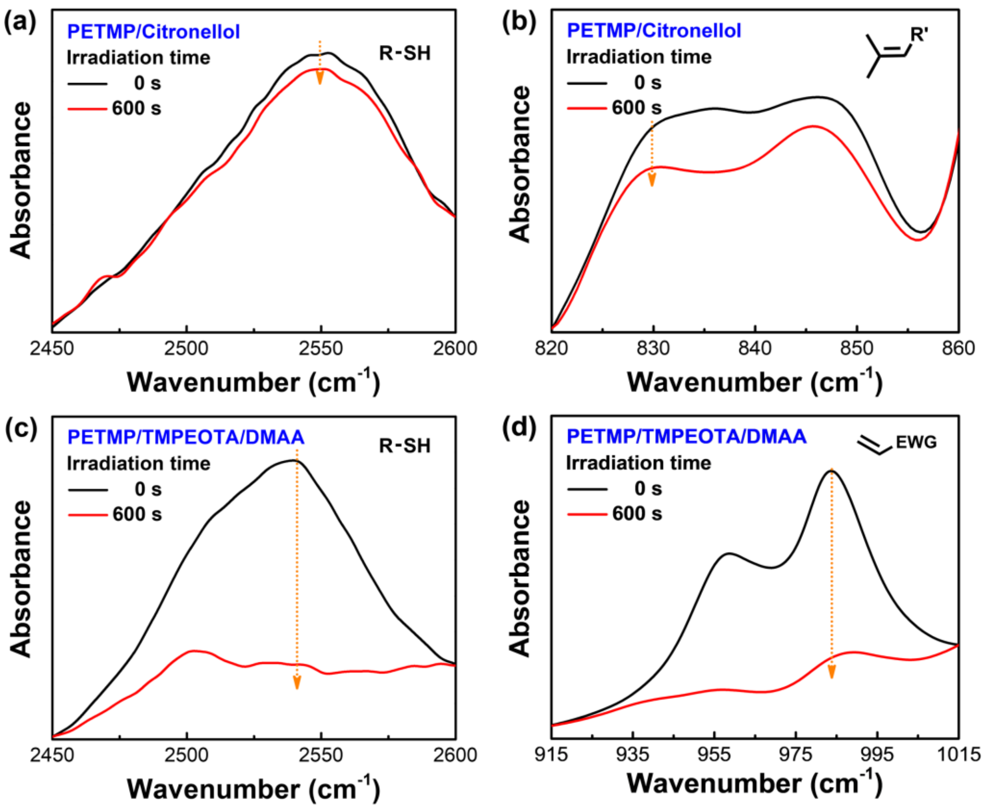

Figure S3. Representative RT-FTIR spectra of (a,b) the PETMP/citronellol system (functional group ratio: 1/1)[7] and (c,d) the PETMP/TMPEOTA/DMAA system (functional group ratio: 9/6/28), respectively, when irradiated by 38 mW·cm^{-2} of visible light (wavelength: 420~500 nm). In the PETMP/citronellol mixture, 30 wt% of DMF was added as the solvent. 1.0 wt% of RB and 1.3 wt% of NPG were added in each mixture to mediate the photoreaction.

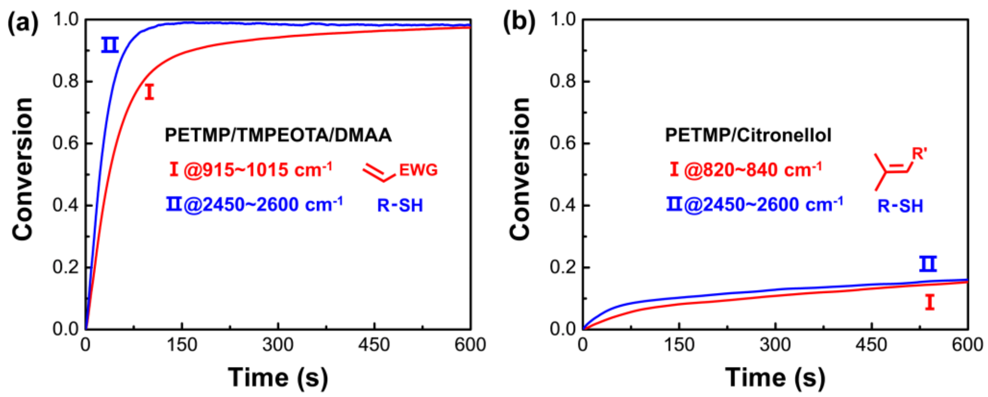

Figure S4. Photoreaction kinetics of the thiol, acryl and citronellyl functional groups in the mixtures of (a) PETMP/TMPEOTA/DMAA (functional group ratio: 9/6/28) and (b) PETMP/citronellol (functional group ratio: 1/1), respectively, when irradiated by 38 mW·cm^{-2} of visible light (wavelength: 420~500 nm). In the PETMP/citronellol mixture, 30 wt% of DMF was added as the solvent. 1.0 wt% of RB and 1.3 wt% of NPG were added in each mixture to mediate the photoreaction.

2.4 Photocyclization of TPE-4CN (the AIEgen) within Holographic Gratings

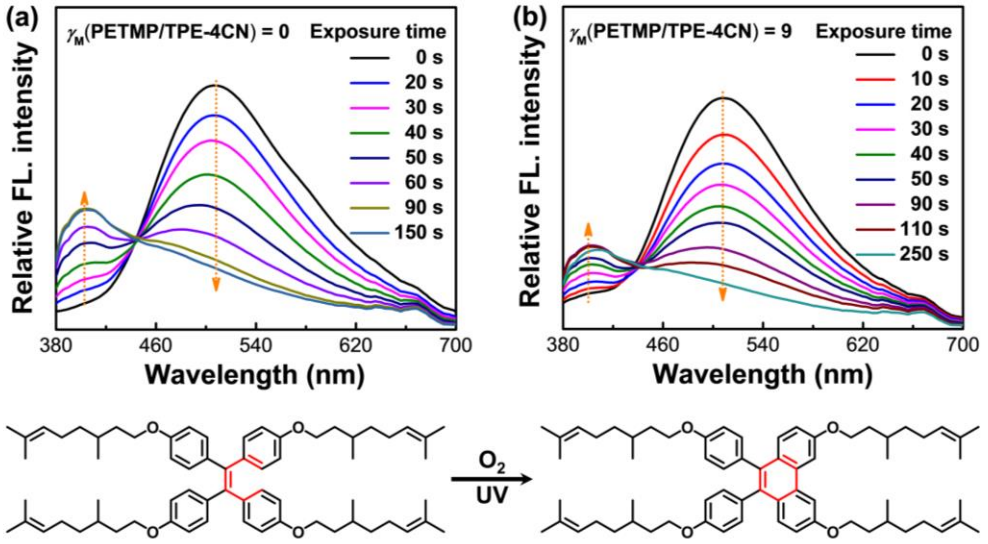

Figure S5. Fluorescent (FL.) spectra of holographic gratings during photocyclization of TPE-4CN upon UV irradiation (365 nm, 100 mW·cm^{-2}). γ_M(PETMP/TPE-4CN) was (a) 0 and (b) 9, respectively. Excitation wavelength: 365 nm. The decreased fluorescence at 508 nm and

increased fluorescence at 403 nm indicating the photocyclization of TPE-4CN and weakened energy transfer from TPE-4CN to the LC.[1]

2.5 Comparison of Thermosensitivity

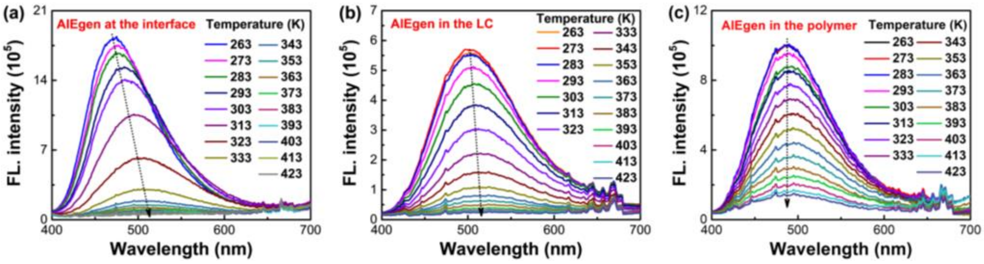

Figure S6. Fluorescence (FL.) intensity against temperature of the AIEgen when (a) bonded at the polymer/LC interface, (b) enriched in the LC-rich phase and (c) cross-linked in the polymer, respectively. Excitation wavelength: 380 nm.

2.6 Fluorescent Emission when Fixing the AIEgen at the Interface

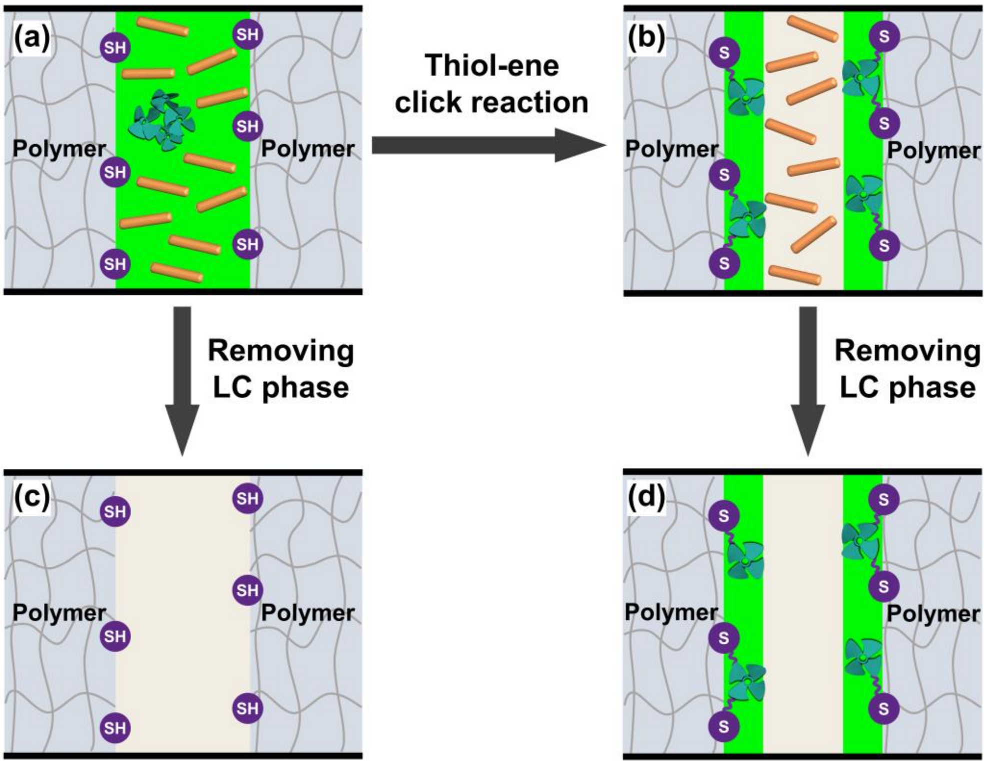

Figure S7. Schematic illustration of the AIEgen when (a) enriched in the LC phase after holographic patterning, and (b) bonded at the polymer/LC interface after thiol-ene click reaction. (a) and (b) are transformed into (c) and (d) after removing the LC by soaking in

n-hexane, respectively.

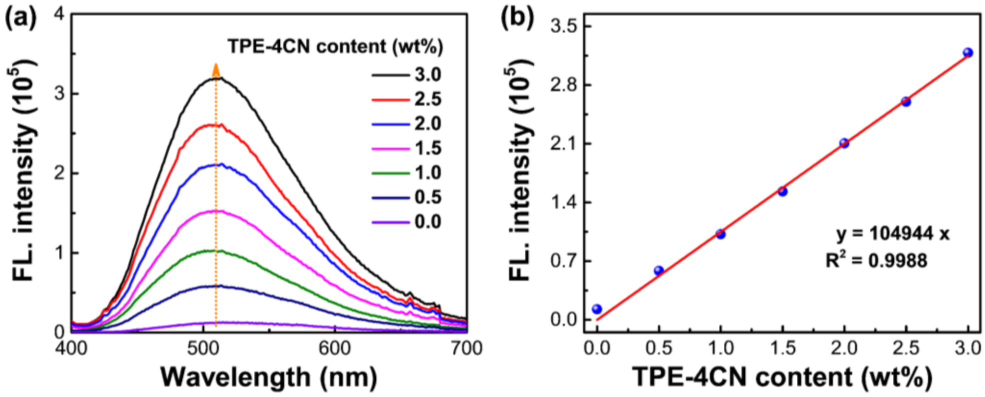

Figure S8. (a) Fluorescence spectra of holographic gratings against the content of the AIEgen (e.g., TPE-4CN) after removing the LC by *n*-hexane. Excitation wavelength: 380 nm. The content of PETMP was 13.7 wt%. (b) Fluorescent (FL.) intensity@510 nm of holographic gratings against TPE-4CN content, indicating the chemical fixation of TPE-4CN via thiol-ene click reaction. Testing level for statistical analysis: $p < 0.05$.

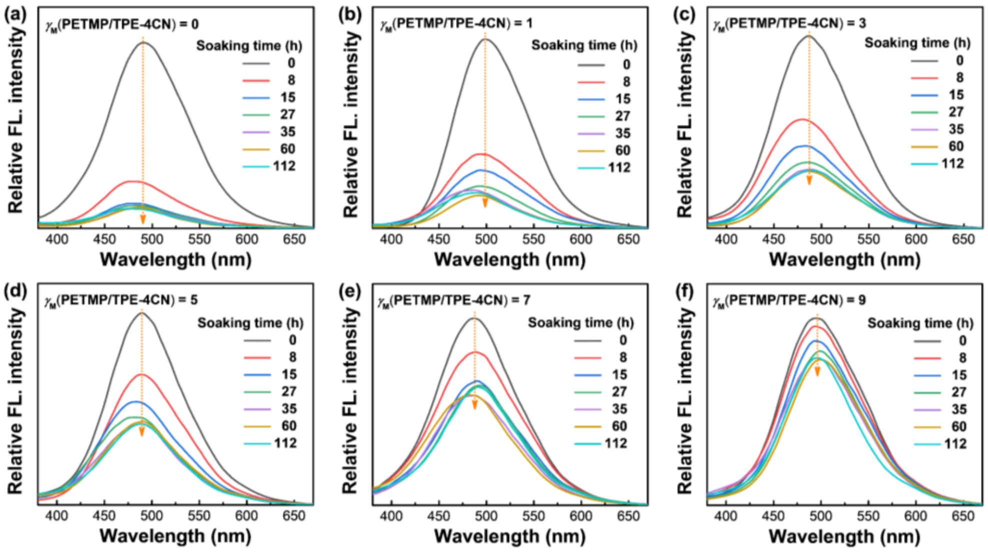

Figure S9. Fluorescence spectra (excitation@365 nm) of holographic gratings against soaking time in *n*-hexane when varying γ_M(PETMP/TPE-4CN): (a) 0, (b) 1, (c) 3, (d) 5, (e) 7, (f) 9, indicating that more thiols were able to fix more TPE-4CN molecules at the interface via thiol-ene click reaction and to prevent solvent extraction.

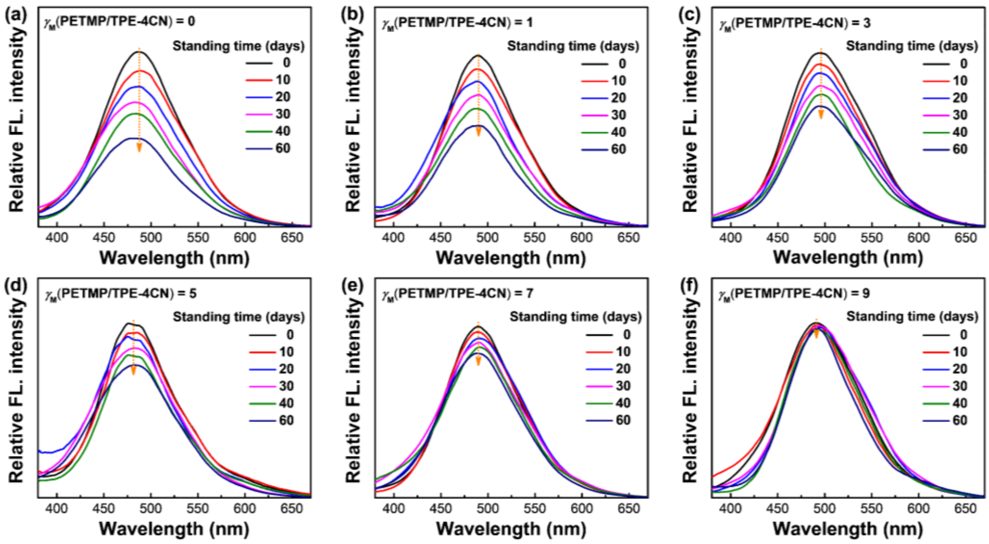

Figure S10. Fluorescence spectra (excitation@365 nm) of holographic gratings against standing time in dark when varying γ_M(PETMP/TPE-4CN): (a) 0, (b) 1, (c) 3, (d) 5, (e) 7, (f) 9, indicating that more thiols were able to fix more TPE-4CN molecules at the interface via thiol-ene click reaction and to prevent TPE-4CN diffusion.

2.7 Characterization of Anionic Reaction Products

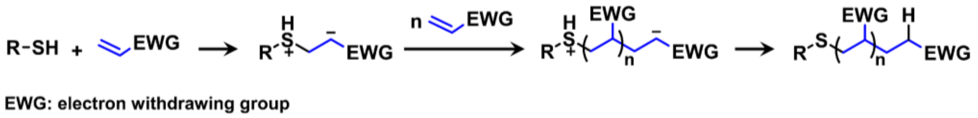

Figure S11. Proposed mechanism of the thiol-based anionic reaction.

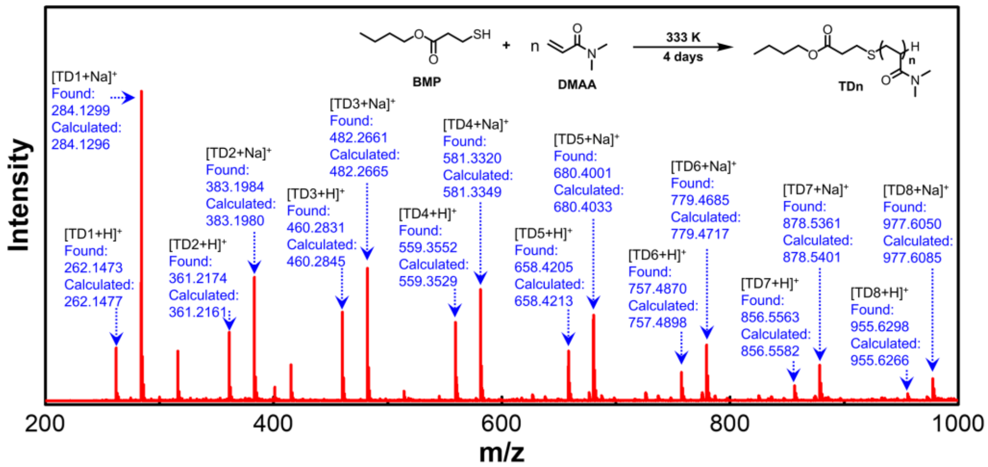

Figure S12. HRMS of oligomers formed by thiol-based anionic reaction of DMAA when initiated by BMP. The molar ratio of DMAA to BMP was 4 prior to reaction. The reaction was maintained for 4 days at 333 K.

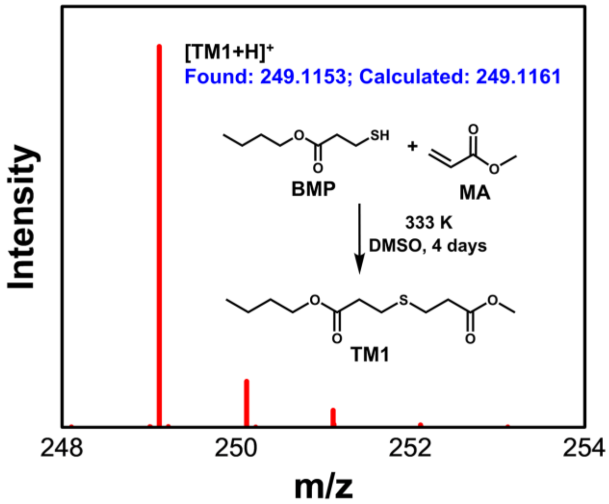

Figure S13. HRMS of thioether formed by anionic reaction between BMP and MA. The molar ratio of MA to BMP was 4 prior to reaction. The solvent content (e.g., DMSO) was 30 wt% and the reaction was maintained for 4 days at 333 K.

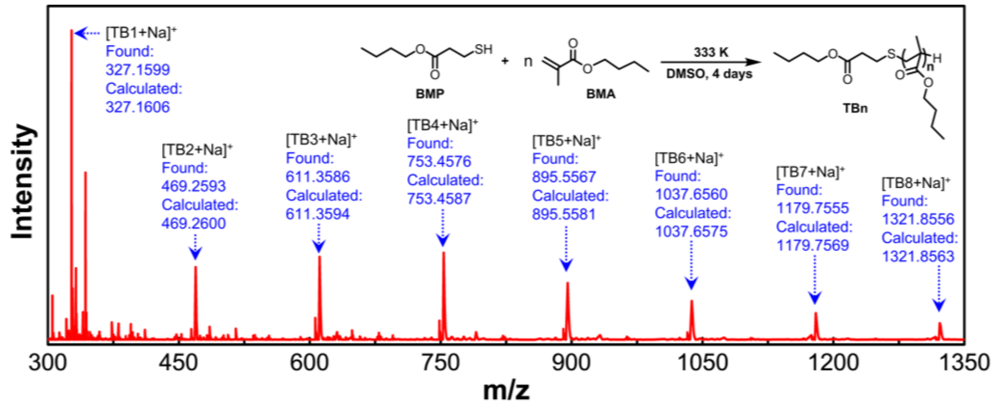

Figure S14. HRMS of oligomers formed by thiol-based anionic reaction of BMA when initiated by BMP. The molar ratio of BMA to BMP was 4 prior to reaction and the solvent content (e.g., DMSO) was 30 wt%. The reaction was maintained for 4 days at 333 K.

2.8 Polarized Optical Microscopy and Confocal Images

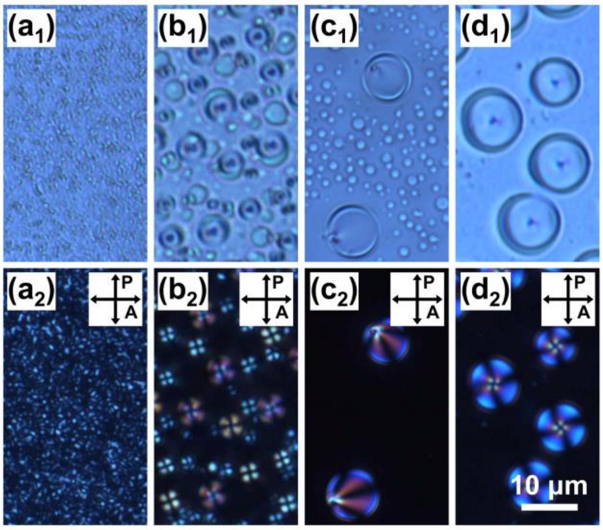

Figure S15. Optical microscopy images of polymer/LC composites with different thiols: (a_1,a_2) BMP, (b_1,b_2) EGBMP, (c_1,c_2) TMPTMP and (d_1,d_2) PETMP. (a_1~d_1) Bright-field and (a_2~d_2) polarized optical microscopy images were captured at room temperature. The functional group ratio of thiol to TPE-4CN was 9. Sample thickness: 3 μm.

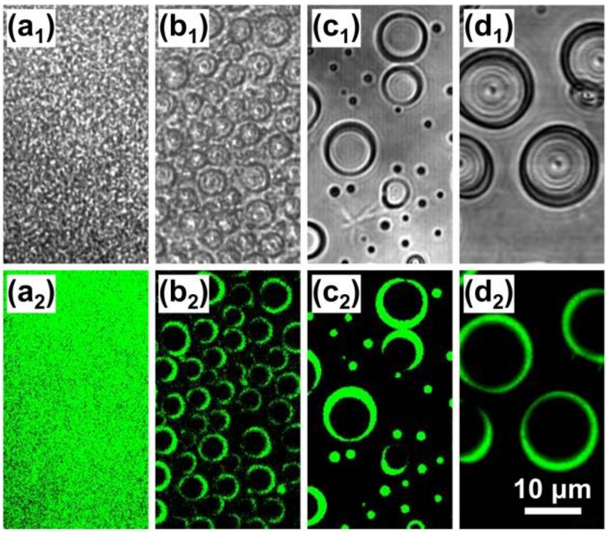

Figure S16. Confocal images of polymer/LC composites with different thiols: (a_1,a_2) BMP, (b_1,b_2) EGBMP, (c_1,c_2) TMPTMP and (d_1,d_2) PETMP. (a_1~d_1) Bright-field and (a_2~d_2) fluorescent images were captured at room temperature. The functional group ratio of thiol to TPE-4CN was 9. Fluorescent images were excited by a 405 nm laser to prevent the excitation of the LC (e.g., P0616A). Sample thickness: 3 μm.

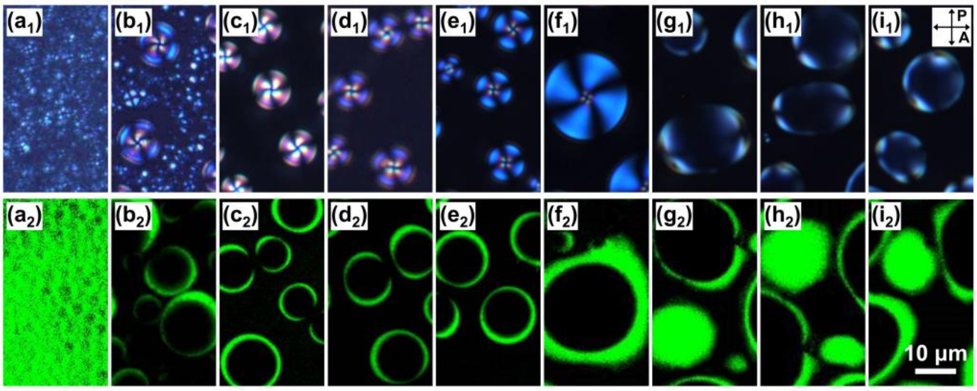

Figure S17. Polarized optical microscopy (a_1~i_1) and confocal images (a_2~i_2) of polymer/LC composites formed via sequential anionic reaction induced phase separation and photo-mediated thiol-ene click reaction. Anionic reaction time was: (a_1,a_2) 0 h, (b_1,b_2) 6 h, (c_1,c_2) 12 h, (d_1,d_2) 18 h, (e_1,e_2) 24 h, (f_1,f_2) 48 h, (g_1,g_2) 72 h, (h_1,h_2) 96 h and (i_1,i_2) 120 h. γ_M(PETMP/TPE-4CN) was 9. Fluorescent images were excited by a 405 nm laser to prevent the excitation of the LC (e.g., P0616A). Sample thickness: 3 μm.

2.9 Effect of Anionic Reaction Time (ART) on the Holographic Performance

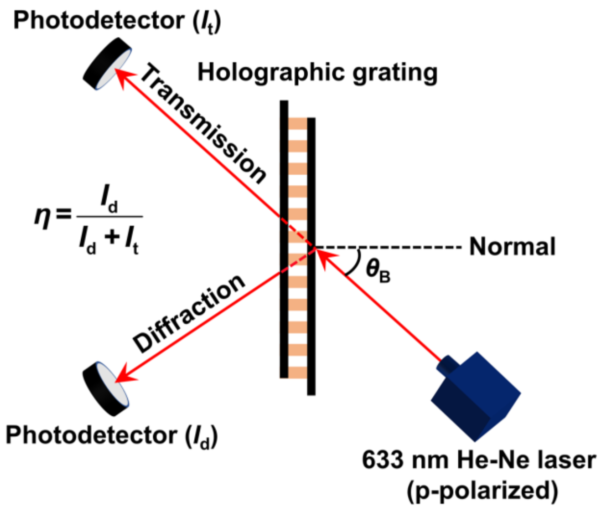

Figure S18. Schematic diagram for measuring the diffraction efficiency (η) that was defined as the intensity ratio of diffraction to the sum of diffraction and transmission at the Bragg angle (θ_B).

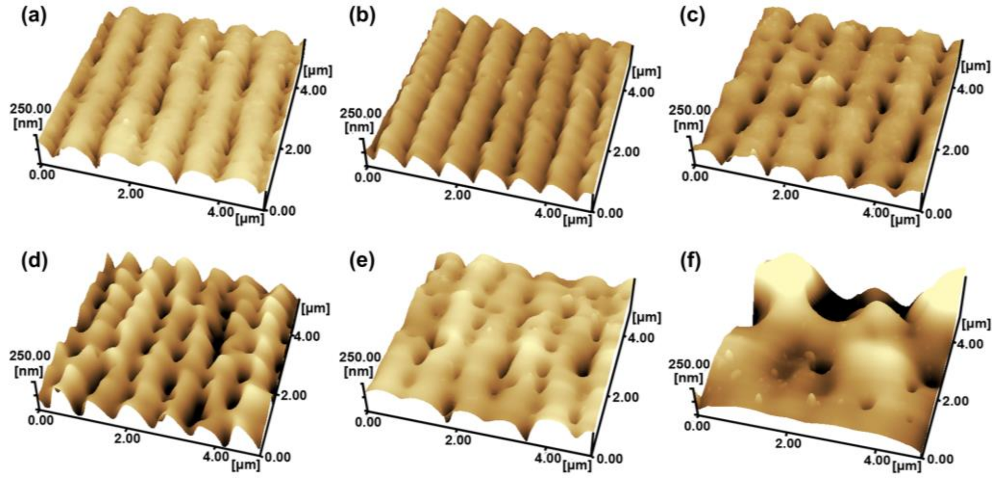

Figure S19. AFM images of holographic gratings formed after anionic reaction with different ART: (a) 0, (b) 1, (c) 2, (d) 3, (e) 4, (f) 5 h. AFM characterizations were conducted after removing the LC by *n*-hexane. γ_M(PETMP/TPE-4CN) was 9. The grating depth was determined to be (a) 102 ± 15, (b) 100 ± 16, (c) 126 ± 31, (d) 155 ± 36, (e) 131 ± 29 and (f) 29 ± 25 nm, respectively.

2.10 Effect of Thiol Functionality on the Holographic Performance

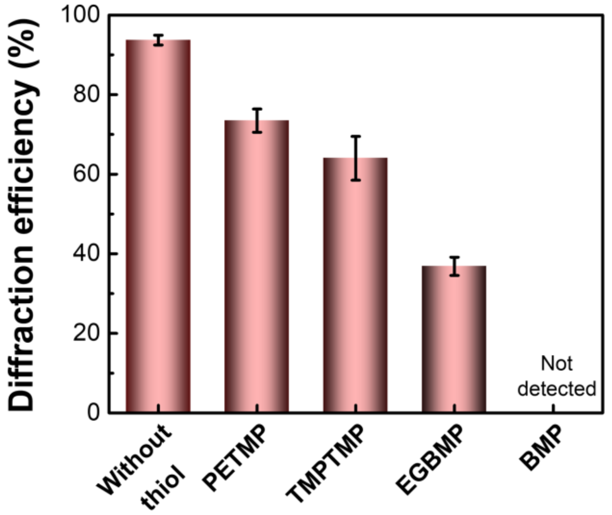

Figure S20. Diffraction efficiencies of holographic gratings when incorporating thiols with different functionalities at the ART of 0 h. The functional group ratio of thiol to citronellyl was 9/1.

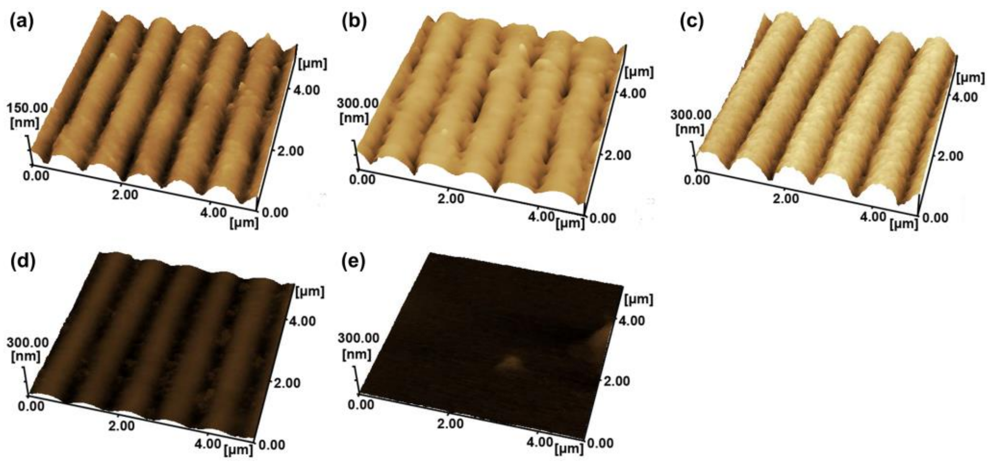

Figure S21. AFM images of holographic gratings with different thiols: (a) without thiol, (b) PETMP, (c) TMPTMP, (d) EGBMP and (e) BMP. ART was 0 h. AFM characterizations were conducted after removing the LC by *n*-hexane. The functional group ratio of thiol to citronellyl was 9/1. The grating depth was determined to be (a) 61 ± 5, (b) 102 ± 15, (c) 174 ± 19, (d) 45 ± 4 nm and (e) unmeasurable, respectively.

2.11 Effect of Thiol Content on the Holographic Performance

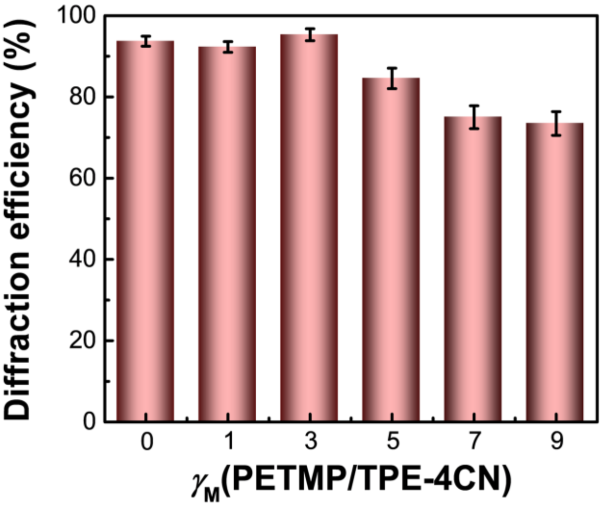

Figure S22. Diffraction efficiency of holographic gratings against γ_M(PETMP/TPE-4CN) at ART of 0 h.

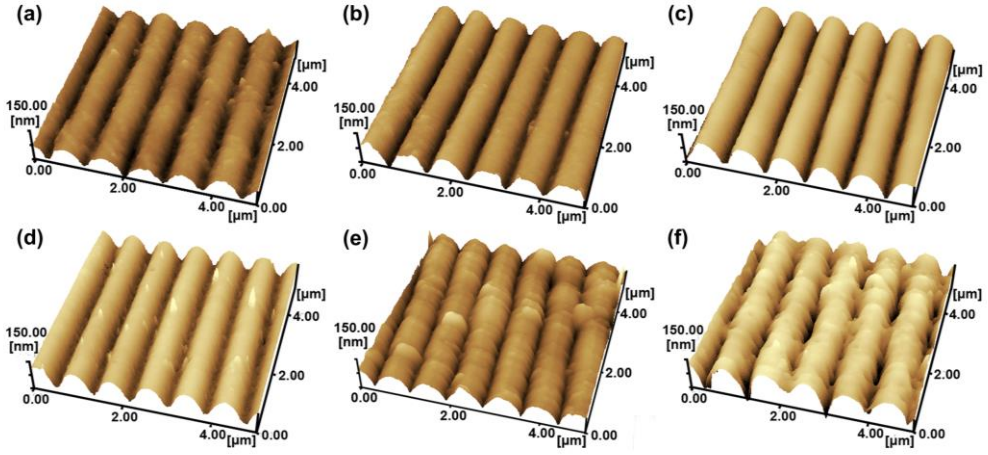

Figure S23. AFM images of holographic gratings at the ART of 0 h with varied γ_M(PETMP/TPE-4CN): (a) 0, (b) 1, (c) 3, (d) 5, (e) 7, (f) 9. AFM characterizations were conducted after removing the LC by *n*-hexane. The grating depth was determined to be (a) 61 ± 5, (b) 80 ± 5, (c) 96 ± 3, (d) 87 ± 5, (e) 65 ± 7 and (f) 102 ± 15 nm, respectively.

2.12 Effect of Thiol Content on the Fluorescent Emission

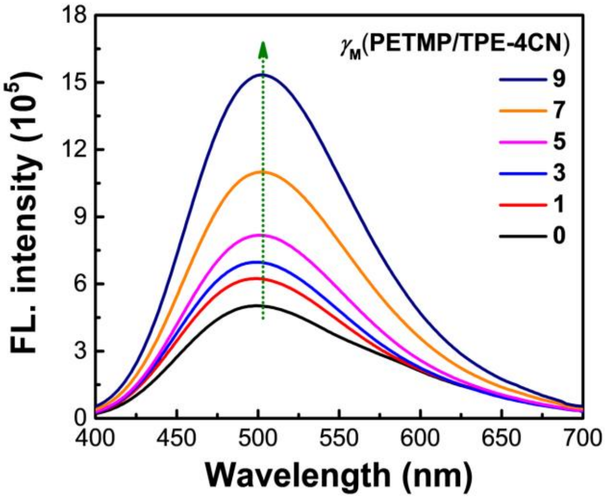

Figure S24. Fluorescence (FL.) intensity of holographic gratings against γ_M(PETMP/TPE-4CN).

2.13 Effect of ART on the Photoreaction Kinetics

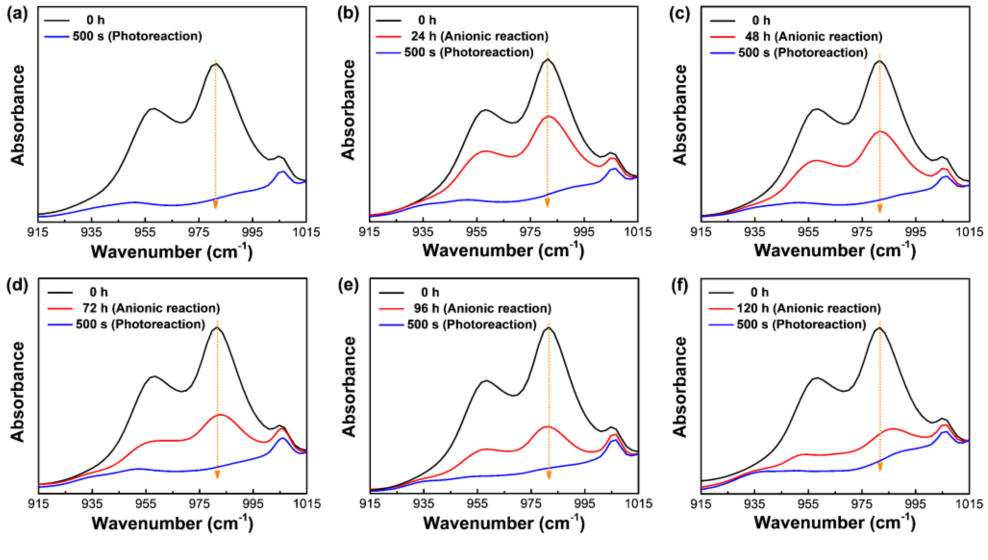

Figure S25. Representative RT-FTIR spectra of the acryl functional group after anionic reaction and photoreaction. The functional group ratio of PETMP, TMPEOTA, DMAA and TPE-4CN was 9/6/28/1.

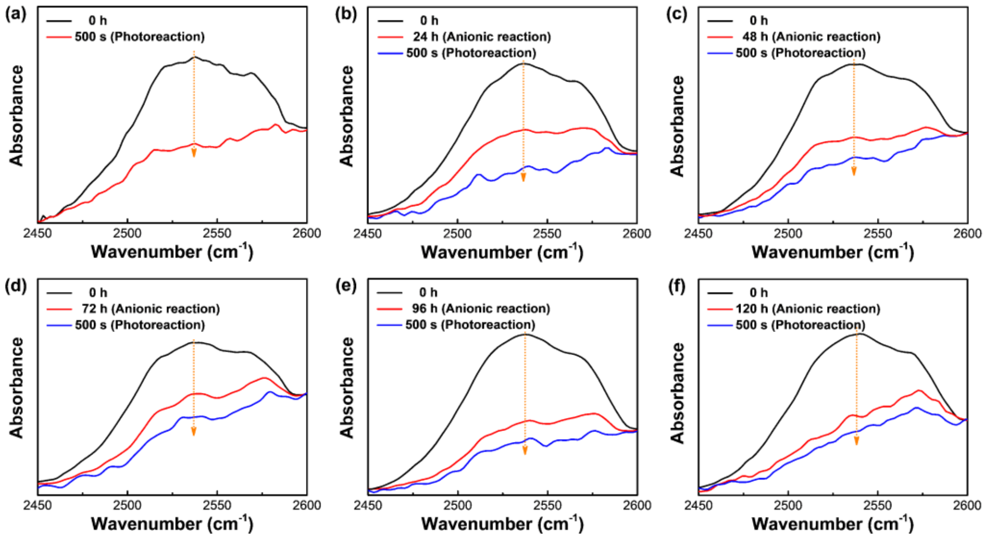

Figure S26. Representative RT-FTIR spectra of the thiol functional group after anionic reaction and photoreaction. The functional group ratio of PETMP, TMPEOTA, DMAA and TPE-4CN was 9/6/28/1.

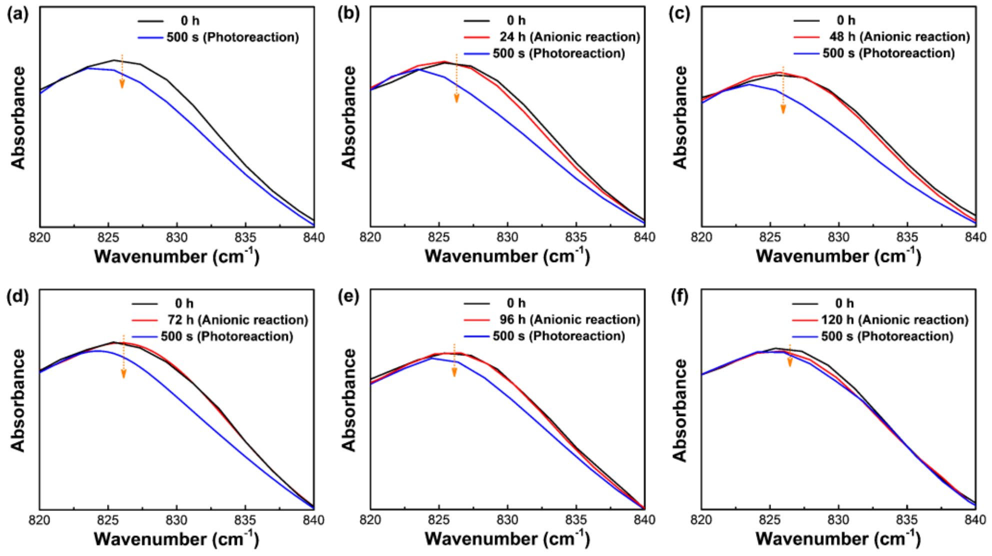

Figure S27. Representative RT-FTIR spectra of the citronellyl functional group after anionic reaction and photoreactiom. The functional group ratio of PETMP, TMPEOTA, DMAA and TPE-4CN was 9/6/28/1.

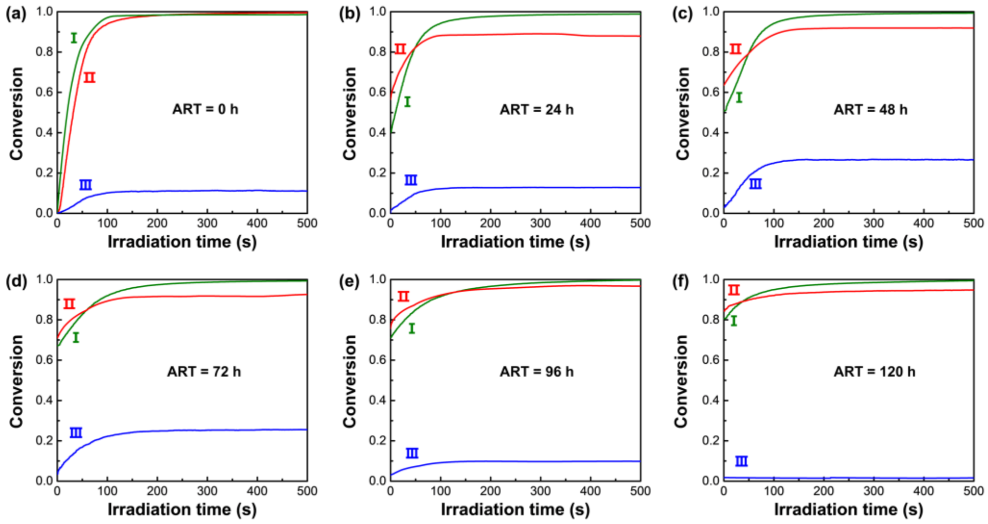

Figure S28. Photoreaction kinetics of (I) acryl, (II) thiol and (III) citronellyl functional groups in the holographic mixture when varying ART: (a) 0, (b) 24, (c) 48, (d) 72, (e) 96 and (f) 120 h, respectively. Samples were exposed by 420~500 nm light for 500 s at an intensity of 38 mW·cm^{-2}. The functional group ratio of PETMP, TMPEOTA, DMAA and TPE-4CN was 9/6/28/1.

3. References

[1] Y. Zhao, X. Y. Zhao, M.-D. Li, Z. A. Li, H. Y. Peng, X. L. Xie, *Angew. Chem., Int. Ed.* **2020**, *59*, 10066.

[2] a) X. Y. Zhao, S. S. Sun, Y. Zhao, R.-Z. Liao, M.-D. Li, Y. G. Liao, H. Y. Peng, X. L. Xie, *Sci. China Mater.* **2019**, *62*, 1921; b) H. Y. Peng, D. P. Nair, B. A. Kowalski, W. X. Xi, T. Gong, C. Wang, M. Cole, N. B. Cramer, X. L. Xie, R. R. McLeod, C. N. Bowman, *Macromolecules* **2014**, *47*, 2306.

[3] H. Y. Peng, S. G. Bi, M. L. Ni, X. L. Xie, Y. G. Liao, X. P. Zhou, Z. G. Xue, J. T. Zhu, Y. Wei, C. N. Bowman, Y. W. Mai, *J. Am. Chem. Soc.* **2014**, *136*, 8855.

[4] a) X. M. Zhang, W. J. Yao, X. P. Zhou, W. Wu, Q. K. Liu, H. Y. Peng, J. T. Zhu, I. I. Smalyukh, X. L. Xie, *Compos. Sci. Technol.* **2019**, *181*, 107705; b) W. Luo, M. L. Ni, X. P. Zhou, H. Y. Peng, X. L. Xie, *Composites, Part B* **2020**, *199*, 108290.

[5] H. Y. Peng, L. Yu, G. N. Chen, Z. G. Xue, Y. G. Liao, J. T. Zhu, X. L. Xie, I. I. Smalyukh, Y. Wei, *ACS Appl. Mater. Interfaces* **2019**, *11*, 8612.

[6] a) C. E. Hoyle, C. N. Bowman, *Angew. Chem., Int. Ed.* **2010**, *49*, 1540; b) B. D. Fairbanks, L. J. Macdougall, S. Mavila, J. Sinha, B. E. Kirkpatrick, K. S. Anseth, C. N. Bowman, *Chem. Rev.* **2021**, *121*, 6915.

[7] a) C. D. Warren, R. W. Jeanloz, *Biochemistry* **1973**, *12*, 5038; b) A. S. Quick, J. Fischer, B. Richter, T. Pauloehrl, V. Trouillet, M. Wegener, C. Barner-Kowollik, *Macromol. Rapid Commun.* **2013**, *34*, 335.
